# Factors influencing the efficiency of cellphone-based CBT for treating sleep disorders

**DOI:** 10.3389/fpsyt.2022.974888

**Published:** 2022-10-10

**Authors:** Nannan Hu, You Xu, Hongjing Mao

**Affiliations:** Affiliated Mental Health Center and Hangzhou Seventh People's Hospital, Zhejiang University School of Medicine, Hangzhou, China

**Keywords:** insomnia, CBT-I, effective factors, online, PSQI

## Abstract

**Objective:**

This survey aimed to better comprehend the factors influencing patient response to insomnia treatment.

**Methods:**

We conducted an online survey. A total of 1,395 patients completed the questionnaire at baseline. Insomnia, anxiety and depressive symptoms were evaluated using the Pittsburgh Sleep Quality Index (PSQI), 7-item Generalized Anxiety Disorder assessment (GAD-7) and 9-item Patient Health Questionnaire (PHQ-9), respectively. A total of 488 patients completed at least two surveys (baseline and monthly surveys thereafter) and reported that the online CBT was effective at the 1-year follow-up. The 488 patients were divided into three groups: the rapid (treatment effective at 4 weeks), intermediate (4–16 weeks), and delayed-response group (over 16 weeks).

**Results:**

Analysis of the demographic characteristics of the 488 patients did not reveal significant sex differences among the three groups (*P* = 0.111). However, the groups significantly differed in age (*P* = 0.001) and education (*P* = 0.006). Compared to the rapid response group, the delayed-response group had a higher mean age (*P* < 0.01) and a slightly lower level of education. The duration of the disorder was longer in the delayed-response group. Multivariate logistic regression showed that male sex, junior high school education, and higher PSQI were independent risk factors for the delayed response to treatment.

**Conclusion:**

Many factors affected the efficiency of insomnia treatment. Male sex, junior school education, and a high PSQI score predicted delayed response to insomnia treatment.

## Introduction

Insomnia is the most common sleep disorder, affecting 10–25% of adults in most countries (1). Insomnia imposes a psychological and financial burden on individuals and society; This disorder is associated with an increased risk of illness, such as heart attack, depression, anxiety, stroke, or hypertension (2–5).

Insomnia treatments are mainly divided into drug treatments, psychotherapy, and physical therapy. Drug treatments include benzodiazepines, antihistamines, and antipsychotics. In most cases, benzodiazepines are effective for facilitating sleep. In terms of psychotherapy, cognitive behavioral therapy (CBT), has been widely used to treat insomnia. Physical treatments for insomnia currently under investigation include transcranial magnetic stimulation (6).

At present, the main non-drug treatment for insomnia is CBT for insomnia (CBT-I), which focus on changing bad sleep habits by, providing sleep hygiene education, changing negative thoughts, attitudes, and beliefs about sleep, and using a series of behavioral methods to improve sleep (7). CBT-I incorporates findings from studies of sleep hygiene, stimulation control therapy, sleep restriction therapy, cognitive therapy, and relaxation therapy (7). This insomnia treatment is effective (8). However, the patient response to CBT-I is slow. Additionally, CBT is time consuming and labor-intensive, with high demands for personnel, space, time, etc. Therefore, the implantation of CBT in clinical practice is often hindered by insufficient medical resources or poor patient compliance.

Internet-based CBT-I provides additional mobility, allowing patients to receive CBT-I at any time and in any place through a mobile phone application, reducing labor and material resources. Compared to other non-CBT treatments, online CBT better improves sleep efficiency, reduces fatigue, improves mood, and improves overall daytime functioning (9–12). Internet-based CBT has the potential to be used in primary care institutions (13) and is as efficacious as face-to-face CBT (14). The present study was a real-world study that examined the factors surrounding the efficiency of online CBT plus pharmacotherapy. According to their speed of response to insomnia treatment, patients were divided into three groups: the rapid, intermediate- and delayed response groups. We then identify factors associated with the speed of response among three groups of insomnia patients.

## Materials and methods

### Ethics approval

The Ethics Committee of the Institutional Review Board of Hangzhou Seventh's Hospital approved this study. All participants provided written informed consent. All procedures were performed in accordance with relevant guidelines.

### Participants

Outpatients of Hangzhou Seventh People's Hospital were enrolled from January 1, 2017 to September 30, 2020. Participants were instructed to keep a sleep diary and undergo online CBT through the “Good Sleep 365” platform. “Good Sleep 365” is a cellphone app for insomnia patients that allows patients to keep a sleep diary, undergo evaluation in the app. The online CBT included sleep restriction, stimulus control, relaxation training, and cognitive reappraisal.

### Inclusion and exclusion criteria

The following inclusion criteria were applied to patients: (1) insomnia [according to the criteria of the International Statistical Classification of Diseases and Related Health Problems, 10th edition (ICD-10)] lasting at least 1 month, and (2) age in the range of 18–65 years. A total of 1,395 patients had a Pittsburgh Sleep Quality Index (PSQI) score >9 at baseline, indicating moderate or severe insomnia, and were followed up at least once. The patients underwent online CBT combined with drug treatment. After 1 year followed-up, 488 patients responded to treatment (PSQI < 9 on at least two assessments). Patients were excluded if they met any of the following criteria: (1) severe physical disease; (2) alcohol/drug abuse or dependence; or (3) women who were pregnant, lactating, or experiencing their menstrual period.

### Study design

Our study used self-reported questionnaires. All participants completed the questionnaires and received online CBT through the app. The online CBT included sleep restriction, stimulus control, cognitive reappraisal, sleep hygiene education, relaxation training and other CBT-I methods and skills. The app also provided information about insomnia, its impacts, and how to prevent its recurrence. This information was presented to the patients in the form of video or audio recordings. After the patient completed the daily sleep diary, the platform provided a new recording (62 videos and 4 audio recordings providing: relaxation training instruction); patients could also repeat previous content.

The following questionnaires were administered at baseline and monthly thereafter to evaluate mental health.

Demographic questionnaire. This questionnaire collected information on patient sex, age, marital status and education level.

The Pittsburgh Sleep Quality Index (PSQI). The PSQI is a self-rating scale with 19 items and a total score of 21 points. A score of 5 points or higher indicates low-quality sleep (15). PSQI scores can also be used to classify insomnia: 0–4 indicates no insomnia; 5–9 indicates mild insomnia; 10–14 indicates moderate insomnia, and 15–21 indicates severe insomnia.

The 7-item Generalized Anxiety Disorder assessment (GAD-7). The GAD-7 is used to assess the severity of anxiety (16) and has good reliability and validity (17). The GAD-7 has seven items, with a total score of 21 points. GAD-7 scores can be used to classify the severity of anxiety: 0–4 indicates no anxiety; 5–9 indicates mild anxiety; 10–14 indicates moderate anxiety, and 15–21 indicates severe anxiety.

The 9-item Patient Health Questionnaire (PHQ-9). The PHQ-9 has 9 items and mainly evaluates depressive symptoms (18), with a total score of 21 points. Higher PHQ-9 scores indicate more severe depressive symptoms. 0–4 indicates no depressive symptoms; 5–9 indicates mild depression; 10–14 indicates moderate depression, and 15–21 indicates severe depression.

### Statistical analysis

Data were analyzed with SPSS version 25.0 (IBM Corp), and a significance level of 0.05. We compared the three groups at baseline. Missing data were inputted by using the last observation carried forward (LOCF) method. Analyses of variance (ANOVAs) were used to compare the three groups. Data are expressed as numbers and percentages. To identify potential risk factors for delayed response to insomnia treatment, multivariate logistic regression analysis was performed. After adjusting for sex, age, and educational level, the associations between the risk factors and outcomes are presented as odds ratios (ORs) and 95% confidence intervals (CIs).

## Results

### Demographic characteristics

First, we assessed the demographic data of the 488 patients in whom treatment was effective. The timing of treatment response was divided into quartiles, forming the following three groups: the rapid- (treatment effective within 4 weeks; 135 patients), intermediate group (treatment effective in 4–16 weeks; 115 patients) and delayed-response group (treatment effective after more than 16 weeks; 238 patients). There was no significant sex difference among the three groups (*P* = 0.111). However, the delayed-response group contained a higher percentage of males (29.8%) than the rapid-and intermediate-response groups (22.2 and 20.9%, respectively). There was no significant difference in the duration of insomnia among the three groups (*P* = 0.185; Table 1). However, the three groups significantly differed in age (*P* = 0.001) and education (*P* = 0.006). Specifically, the delayed-response group had a higher percentage of patients aged >50 years than the repaid-response group, implying that the delayed-response group had a higher mean age than the rapid-response group. The percentage of patients with an education level above college in the rapid-response group was higher than that in the delayed-response group, implying that the rapid-response group had a higher education level than the delayed-response group.

**Table 1:** Demographic characteristics of participants.

**Characteristic**	**Rapid response**,	**Intermediate**	**Delayed response**,	***P*-value**
	***N* (%)**	**response, *N* (%)**	***N* (%)**	
**Sex**				*P* = 0.111
Male	30 (22.2)	24 (20.9)	71 (29.8)	
Female	105 (77.7)	91 (79.1)	167 (70.2)	
**Age, year**	45.50 ± 10.92	47.82 ± 11.66	49.68 ± 10.72	*P* = 0.002^*^
<20	1 (0.8)	0 (0)	2 (0.8)	
20–30	11 (8.4)	7 (6.1)	12 (5.1)	
30–40	33 (25.2)	27 (23.5)	31 (13.1)	
40–50	40 (30.5)	33 (28.7)	73 (30.8)	
51–60	37 (28.2)	29 (25.2)	87 (36.7)	
>60	9 (6.9)	19 (16.5)	32 (13.5)	
**Education level**				*P* = 0.006^*^
Primary school	13 (9.9)	20 (17.4)	33 (13.9)	
Junior high school	18 (13.7)	21 (18.3)	61 (25.7)	
Senior high school	34 (26.0)	26 (22.6)	69 (29.1)	
College	55 (42.0)	45 (39.1)	65 (27.4)	
≥Postgraduate	11 (4.6)	3 (2.6)	9 (3.8)	
**Duration of disorder**				*P* = 0.185
<3 months	39 (29.8)	30 (30.0)	52 (21.9)	
3 months−1 year	24 (18.3)	23 (23.0)	47 (19.8)	
1 year−3 years	24 (18.3)	19 (19.0)	39 (16.5)	
3–5 years	15 (11.5)	14 (14.0)	29 (12.2)	
>5 years	29 (22.1)	29 (29.0)	70 (29.5)	

* P < 0.05.

### Comparison of baseline data

The PSQI scores of the rapid-, intermediate-, and delayed-response groups were 13.09 ± 2.44, 14.11 ± 2.61, and 14.82 ± 2.77, respectively. Thus, there was significant difference (*P* < 0.001) at baseline in PSQI scores among the three groups. The GAD-7 scores of the groups were 4.51 ± 3.49, 4.55 ± 3.29, and 4.58 ± 3.33, respectively. There was no significant difference in the GAD-7 scores among the groups (*P* = 0.991). The PHQ-9 scores of the groups were 2.31 ± 1.59, 2.63 ± 1.51, and 2.52 ± 1.71, respectively. There was no significant difference in PHQ-9 scores among the groups (*P* = 0.273). The delayed-response group had higher PSQI scores than the other groups (Table 2).

**Table 2:** Scores of the three groups at baseline [mean ± standard deviation (SD)].

**Scale**	**Rapid response**	**Intermediate response**	**Delayed-response**	***F***	***P*-value**
PSQI (insomnia)	13.09 ± 2.44	14.11 ± 2.61	14.82 ± 2.77	18.578	<0.001^*^
GAD-7 (anxiety)	4.51 ± 3.49	4.55 ± 3.29	4.58 ± 3.33	0.009	0.991
PHQ-9 (depression)	2.31 ± 1.59	2.63 ± 1.51	2.52 ± 1.71	1.300	0.273

PSQI, 19-item pittsburgh sleep quality index; GAD-7, 7-item generalized anxiety disorder assessment; PHQ-9, 9-item patient health questionnaire.

* P < 0.05.

**Figure 1:**
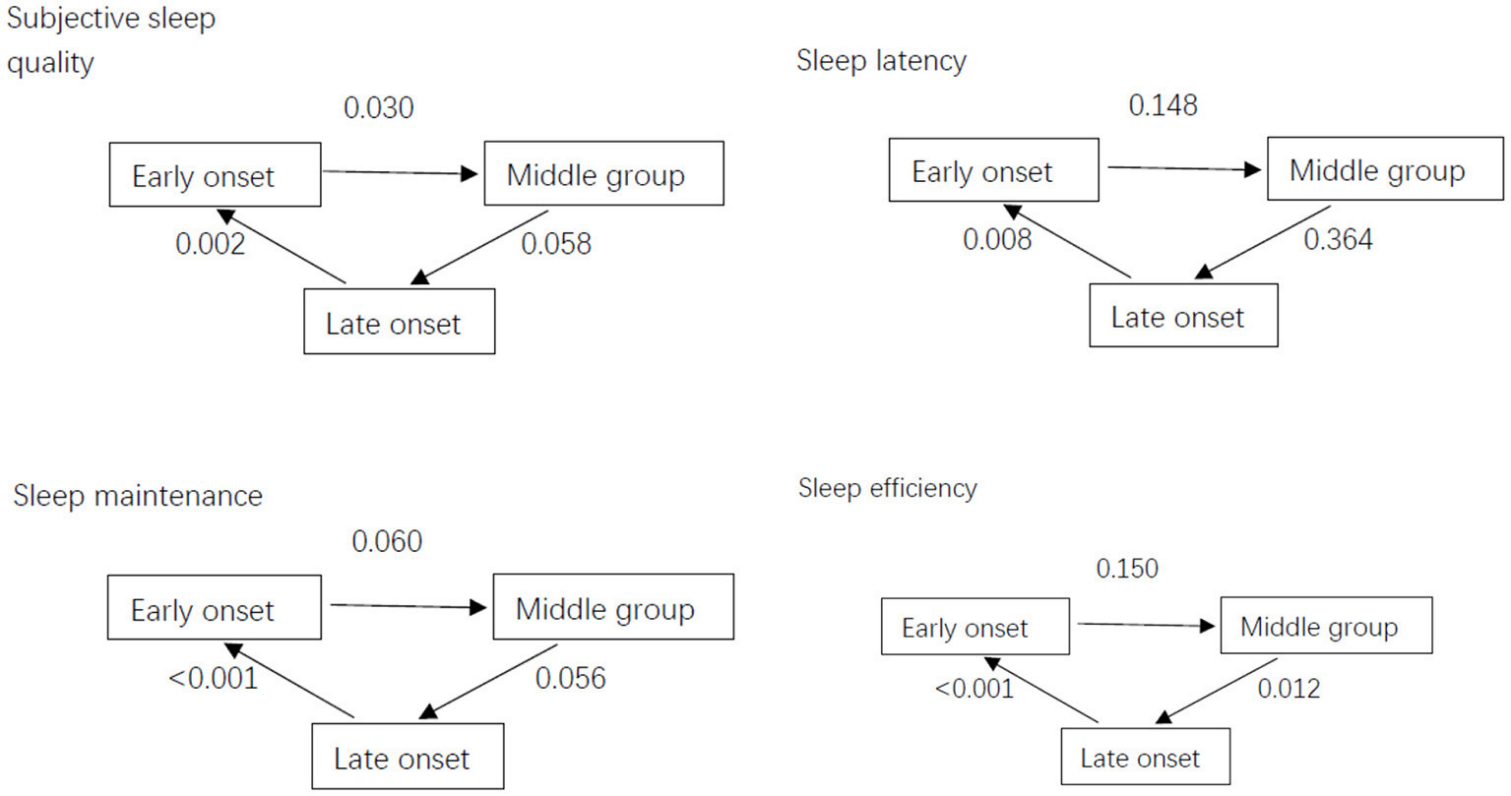
Differences in PSQI factor scores of insomnia patients among the three groups.

The PSQI scale has 7 factors: subjective sleep quality, sleep latency, sleep maintenance, sleep efficiency, sleep disorders, hypnotic drug use and daytime function. There were significant differences in subjective sleep quality (*P* = 0.006), sleep latency (*P* = 0.030), sleep maintenance (< 0.001), and sleep efficiency (< 0.001) among the three groups. The factor scores of the delayed-response group were higher than those of the rapid-response group (Table 3; Figure 1).

**Table 3:** PSQI factors scores of insomnia patients in the three groups at baseline (mean ± SD).

**PSQI factor**	**Rapid response**	**Intermediate response**	**Delayed response**	***F***	***P*-value**
Subjective sleep quality	1.83 ± 0.89	2.06 ± 0.75	2.11 ± 0.84	5.113	0.006^*^
Sleep latency	1.87 ± 0.93	2.04 ± 0.89	2.14 ± 0.93	3.549	0.030^*^
Sleep maintenance	2.11 ± 1.00	2.33 ± 0.95	2.53 ± 0.84	9.120	<0.001^*^
Sleep efficiency	2.04 ± 0.99	2.21 ± 0.94	2.46 ± 0.81	10.139	<0.001^*^
Sleep disorders	1.13 ± 0.43	1.17 ± 0.49	1.20 ± 0.50	1.076	0.342
Hypnotic drug use	2.01 ± 0.30	2.21 ± 1.20	2.27 ± 1.16	2.098	0.124
Daytime function	2.04 ± 0.93	2.10 ± 0.89	2.08 ± 0.97	0.137	0.872

PSQI, pittsburgh sleep quality index;

* represents P < 0.05.

### Risk factors for poor mental health outcomes

Multivariate logistic regression analysis showed that male sex, junior high school education, higher PSQI scores and older age were associated with delayed response to insomnia treatment (male sex: OR: 0.44, 95%CI: 0.03–0.084, *P* = 0.036; junior high school education: OR: 1.04, 95%CI: 0.14–1.95, *P* = 0.024; Table 4).

**Table 4:** Risk factors for delayed response to insomnia treatment according to multivariate logistic regression analysis.

**Variable**	**Adjusted OR (95%)**	***P*-value**
**Sex**		
Male	0.44 (0.03–0.84)	0.036
Female	1 [Reference]	NA
Age	0.11 (0.00–0.03)	0.043
PSQI score	0.18 (0.12–0.25)	<0.001
GAD7 score	−0.03 (−0.09–0.03)	0.275
PHQ9 score	0.07 (−0.06–0.20)	0.243
**Education level**		
Primary school	0.75 (−0.21–1.71)	0.124
Junior high school	1.04 (0.14–1.95)	0.024
Senior high school	0.67 (−0.21–1.54)	0.135
College	0.339 (−0.50–1.18)	0.428
≥Postgraduate	1 [Reference]	NA
**Duration of disorder**		
<3 months	−0.29 (−0.79–0.21)	0.254
3 months−1 year	−0.08 (−0.62–0.45)	0.785
1–3 years	−0.33 (−0.87–0.21)	0.236
3–5 years	−0.11 (−0.72–0.51)	0.731
>5 years	1 [Reference]	NA

PSQI, 19-item pittsburgh sleep quality index; GAD-7, 7-item generalized anxiety disorder assessment; PHQ-9, 9-item patient health questionnaire; NA, not applicable.

## Discussion

Insomnia treatment is complicated and time consuming. Drugs are highly important for insomnia treatment. Benzodiazepines, for example, are effective, but patients are likely to develop a tolerance to them over the long-term use. Combining drug treatment with CBT-I effectively reduces the frequency of hypnotic use (19). Additionally, behavioral intervention is recommended as a first-line treatment (8), as the therapeutic effect of CBT-I has been clearly demonstrated (20, 21). CBT-I mainly targets the factors maintain insomnia, as well as the psychological and behavioral problems that cause insomnia. CBT-I as the first-line choice for the treatment to insomnia (22), has been recommended that all patients with chronic insomnia should receive CBT-I by “Guidelines for Management of Chronic Insomnia in Adults”. However, was access to CBT is often limited by time, and available clinicians trained in these skills; thus, individual, face-to-face CBT-I from professional psychiatrists is often unavailable. Similarly, the success of CBT-I depends on patient' compliance. Thus, the efficacy of individual and face-to-face CBT-I are often affected by follow-up and supervision (23). With the increasing popularity of the internet and smartphones in recent years, it has become possible to administer online CBT-I (24).

Our study mainly focused on 488 patients in whom insomnia treatment was effective. These patients were divided into early-, intermediate- and delayed response groups. Analysis of demographic data revealed that no significant sex difference among the three groups. However, the proportion of males in the delayed-response group was higher than that in the other groups. Multivariate logistic regression analysis showed that, male sex was associated with delayed response to treatment. Specifically, men were 44-fold more likely to have delayed response to treatment compared to women. Thus, male sex was a risk factor for delayed response to insomnia treatment. Multivariate logistic regression suggested that age was an independent risk factor for delay response. A 1-year increase in age increased the probability of delayed response to treatment increased by 11%. Thus, we concluded that as age increased, response to treatment slowed. Multivariate logistic regression analysis showed that attaining only junior high school' education was associated with delayed response to treatment. Patients with junior high school were 1.04-fold more likely to have delayed response to treatment than patients with postgraduate education. Patients with a higher level of education might have a better understanding of online CBT or better compliance. Educational attainment also has protective effects against mental disease, as demonstrated in another study (25). Additionally, multivariate logistic regression analysis indicated that higher PSQI scores were associated with delayed response to treatment. Specifically, a 1-point increase in PSQI scores at baseline increased the probability of delayed response to treatment by 18%. The delay-response group also had higher PSQI scores at baseline than the other groups. Thus, these patients took longer to achieve significant improvement of insomnia, suggesting that the treatment of severe insomnia takes much longer. In general, patients with a longer duration of insomnia are believed to have a slower response to treatment. However, the duration of insomnia was not a risk factor for delayed response to treatment in our study.

A previous study showed that female patients are more likely to be anxious and report poor sleep quality than male (26). In the present study, we found that more female patients were enrolled, consistent with previous research. However, we found that male insomnia patients improved more slowly than female patients. Additionally, patients who attained only junior high school education had a slower response to treatment, which might be related to differences in the understanding of online CBT. Generally, the acceptance of online CBT-I is related to its efficacy. However, in the present study, it was difficult to assess patient' compliance with CBT, which might cause bias.

Subjective sleep quality, sleep latency, sleep maintenance, and sleep efficiency were significantly different among the three groups. The mean score on these factors in the delayed-response group was higher than that in the rapid-response group, implying that the delayed-response group experienced worse sleep in these aspects than the rapid-response group. After treatment, there were no significant group differences in subjective sleep quality and sleep latency. Therefore, online CBT mainly improved these two aspects of sleep quality. Sleep restriction, a common CBT-I method, is related to increased slow-wave activity (27). By increasing the time spent out of bed and awake, sleep restriction enhances sleep drive and regulates sleep rhythm.

In some diseases such as Parkinson's disease (28), cancer (29) and heart disease, sleep disorders are very common. Indeed, a genetic study identified the causal effects of insomnia on depression, diabetes, and cardiovascular disease (25). Intriguingly, CBT-I is effective for treating sleep disorders comorbid with somatic disease (30). CBT-I can also be used to treat insomnia comorbid depression, anxiety, PTSD or substance abuse disorders (31, 32). The use of online CBT-I increased access to treatment for geographically remote patients, and the self-directed CBT-I model that does not require the involvement of therapists (33). A study published in JAMA found that after a 1-year follow-up, online CBT-I improved the vast majority of insomnia patients (34). Patients can receive CBT online, increasing access to treatment by reducing obstacle of a lack of trained clinicians and difficulty accessing treatment due to distance. Therefore, the use of online CBT-I should be promoted.

Previous studies have found that online CBT-I is an effective treatment to insomnia, but few studies have examined the latency of response to treatment.

## Limitation

This study has the following limitations. First, it was limited in scope. Most participants were from Zhejiang Province; Thus, the results may not fully represent other regions. Second, patient compliance was difficult to qualify as the platform had no means of tracking patient compliance. Third, volunteer bias may have been present. Individuals willing to participate in the study may have had worse sleep quality, leading to a high rate of severe insomnia in the study.

## Conclusion

Many factors affect the speed of response to insomnia treatment, but few studies have identified these factors. We found that male sex, junior high school education, and high PSQI scores predicted delayed response to insomnia treatment, allowing prediction of the prognosis of insomnia patients.

## Data availability statement

The original contributions presented in the study are included in the article/supplementary material, further inquiries can be directed to the corresponding author/s.

## Ethics statement

The studies involving human participants were reviewed and approved by Ethics Committee of Hangzhou Seventh People's Hospital. The patients/participants provided their written informed consent to participate in this study.

## Author contributions

Writing of the manuscript: NH. Statistical analysis of data: YX and NH. Concept and design: HM. All authors contributed to the article and approved the submitted version.

## Funding

This study was supported by the Hangzhou Science and Technology Bureau (20191203B119) and the Nature Science Foundation of Zhejiang Province (LDF20H090004).

## Conflict of interest

The authors declare that the research was conducted in the absence of any commercial or financial relationships that could be construed as a potential conflict of interest.

## Publisher's note

All claims expressed in this article are solely those of the authors and do not necessarily represent those of their affiliated organizations, or those of the publisher, the editors and the reviewers. Any product that may be evaluated in this article, or claim that may be made by its manufacturer, is not guaranteed or endorsed by the publisher.

## Abbreviations

PSQI: pittsburgh sleep quality index
GAD-7: 7-item generalized anxiety disorder assessment
PHQ-9: 9-item patient health questionnaire
NA: not applicable
CBT: cognitive behavior therapy.
